# Inhibition of skeletal growth of human prostate cancer by the combination of docetaxel and BKM1644: an aminobisphosphonate derivative

**DOI:** 10.18632/oncotarget.8481

**Published:** 2016-03-30

**Authors:** Shumin Zhang, Lajos Gera, Kenza Mamouni, Xin Li, Zhengjia Chen, Omer Kucuk, Daqing Wu

**Affiliations:** ^1^ Department of Urology, Emory University School of Medicine, Atlanta, GA, USA; ^2^ Department of Biochemistry and Molecular Genetics, University of Colorado Denver, Anschutz Medical Campus, School of Medicine, Aurora, CO, USA; ^3^ Department of Biochemistry and Molecular Biology, Medical College of Georgia and GRU Cancer Center, Augusta University, Augusta, GA, USA; ^4^ Department of Biostatistics and Bioinformatics, Rollins School of Public Health, Emory University, Atlanta, GA, USA; ^5^ Department of Hematology and Medical Oncology, Winship Cancer Institute, Emory University School of Medicine, Atlanta, GA, USA; ^6^ MetCure Therapeutics LLC, Atlanta, GA, USA

**Keywords:** prostate cancer, bone metastasis, survivin inhibitor, docetaxel resistance, preclinical models

## Abstract

Bone metastasis is a major cause of prostate cancer (PCa) morbidity and mortality. Despite some success in transiently controlling clinical symptoms with docetaxel-based therapy, PCa patients become docetaxel-resistant and inevitably progress with no cure. We synthesized an acyl-tyrosine bisphosphonate amide derivative, BKM1644, with the intent of targeting bone metastatic PCa and enhancing docetaxel's efficacy. BKM1644 exhibits potent anti-cancer activity in the NCI-60 panel and effectively inhibits the proliferation of metastatic, castration-resistant PCa (mCRPC) cells, with IC_50_ ranging between 2.1 μM and 6.3 μM. Significantly, BKM1644 sensitizes mCRPC cells to docetaxel treatment. Mice with pre-established C4-2 tumors in the tibia show a marked decrease in serum prostate-specific antigen (control: 173.72 ± 37.52 ng/ml, combined treatment: 64.45 ± 22.19 ng/ml; *p* < 0.0001) and much improved bone architecture after treatment with the combined regimen. Mechanistic studies found that docetaxel temporarily but significantly increases survivin, an anti-apoptotic protein whose overexpression has been correlated with PCa bone metastasis and therapeutic resistance. Intriguingly, BKM1644 effectively inhibits survivin expression, which may antagonize docetaxel-induced survivin in bone metastatic PCa cells. Signal transducer and activator of transcription 3 (Stat3) may be involved in the suppression of survivin transcription by BKM1644, as confirmed by a survivin reporter assay. Collectively, these data indicate that BKM1644 could be a promising small-molecule agent to improve docetaxel efficacy and retard the bone metastatic growth of PCa.

## INTRODUCTION

The American Cancer Society estimated that 180,890 patients will be diagnosed with prostate cancer (PCa) in 2016, and 26,120 will die, mostly from metastatic castration-resistant PCa (mCRPC) [1]. Although current treatments, including chemotherapy using docetaxel and cabazitaxel, initially prolong the median survival by 3∼5 months, patients generally relapse within a year and develop extremely resistant tumors [2, 3]. It is imperative to develop novel strategies to overcome therapeutic resistance and improve the survival of mCRPC patients [4–6].

As one of few “nodal” proteins that intersect with multiple cellular networks in the control of cell fate and mitosis, survivin not only plays a pivotal role in tumorigenesis but also significantly contributes to the late stages of cancer progression, including recurrence, metastasis and therapeutic resistance [7, 8]. Overexpression of survivin has been correlated with high Gleason score, poor clinical outcomes, and resistance to hormonal therapy, chemotherapy and radiation therapy in PCa [9–12]. Recently, studies from us and other groups have demonstrated survivin as an important prognostic biomarker and therapeutic target for mCRPC [13–15]. Survivin-targeted therapy, therefore, could be a promising strategy to enhance standard treatment and improve overall survival of mCRPC patients. Various molecular approaches are currently under development; however, most only exhibit modest benefits in PCa patients with localized and metastatic diseases [16].

We have developed a group of novel small-molecule compounds with the intent of targeting bone metastatic PCa [13, 17]. These compounds consist of F5c-OC2Y, an anti-cancer moiety derived from BKM570 [18–23], and a bisphosphonate amide moiety [24–26]. One of these compounds, BKM1740^a^, effectively inhibited the *in vitro* and *in vivo* growth of bone metastatic PCa cells in mouse models. Significantly, BKM1740 acts as a potent inhibitor of survivin [13, 17]. In this communication, we report BKM1644^b^, a novel BKM1740 analog with broad anti-cancer activity that effectively induces the regression of mCRPC via inhibition of survivin in pre-clinical models.

## RESULTS

### BKM1644 exhibits potent *in vitro* cytotoxicity in human cancer cell lines

**Figure 1:**
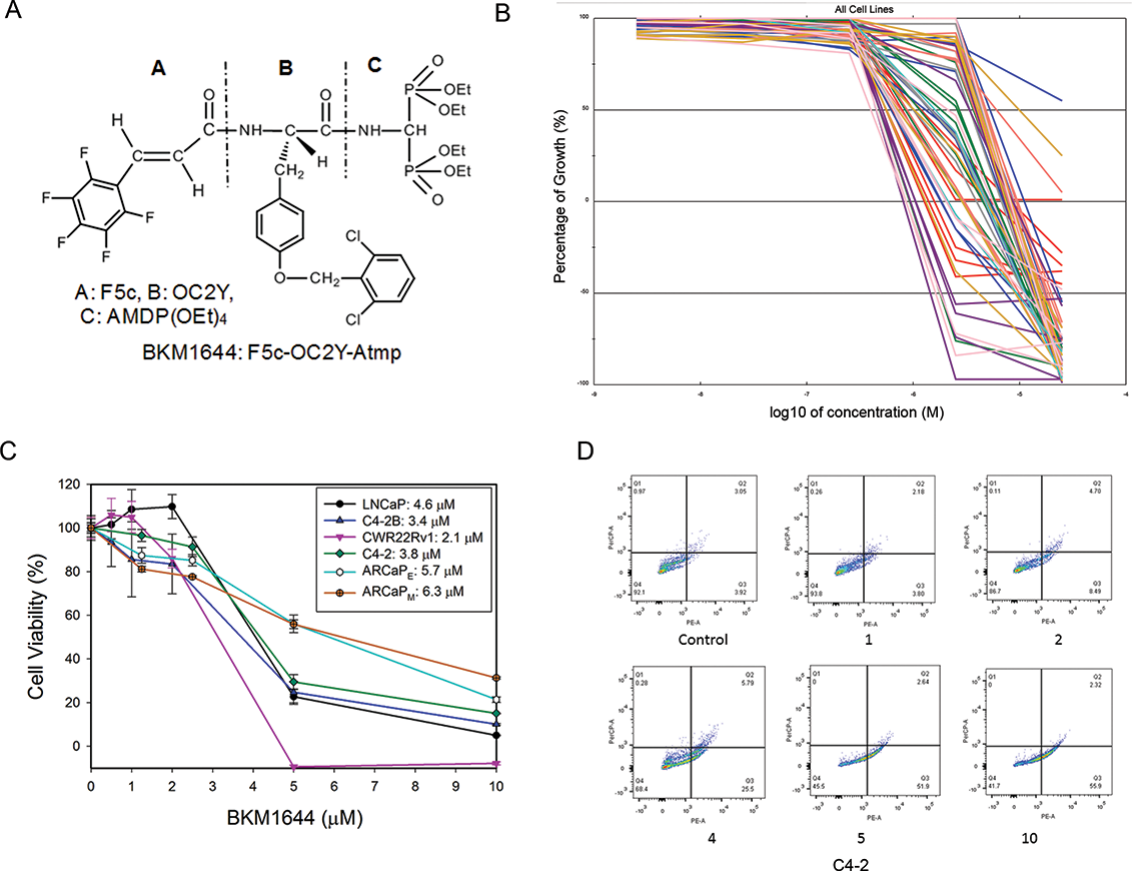
*In vitro* anti-cancer activities of BKM1644 in human cancer cell lines (**A**) Chemical structure of BKM1644. (**B**) Dose response curves of BKM1644 in NCI60 panel. (**C**) *In vitro* cytotoxicity of BKM1644 in a panel of human PCa cell lines (72 h treatment). (**D**) Annexin V expression on C4-2 cells following BKM1644 treatment at the indicated concentrations (μM) (24 h).

By deleting the piperidinyl spacer from BKM1740, we generated BKM1644 [F5c-OC2Y-AMDP(OEt)_4_] (Figure 1A) [17]. Compared to BKM1740, BKM1644 has favorable physicochemical properties as a drug candidate, including a smaller molecular weight (846 Dalton), greater water solubility and easier ability to synthesize. To obtain an unbiased information of the anti-cancer activity of BKM1644, we submitted the compound to the National Cancer Institute Developmental Therapeutics Program and performed NCI-60 screening (Figures 1, S1). BKM1644 induced growth inhibition and cell death across a broad spectrum of human cancer cells. The average GI_50_ (50% growth inhibition) in the NCI-60 panel is 1.4 μM (ranging between 0.8 μM and 10.1 μM), and the average LC_50_ (50% lethal concentration) is 3.0 μM (ranging between 2.9 μM and over 50 μM). In two mCRPC cell lines, PC-3 and DU-145, BKM1644 has LC_50_ values of 3.8 μM and 3.1 μM, respectively (Figure S1). We further examined the *in vitro* cytotoxicity of BKM1644 in other established human PCa cell lines, including LNCaP, C4-2, C4-2B, CWR22Rv1, ARCaP_E_ and ARCaP_M_ cells. The average IC_50_ (50% inhibitory concentration) of BKM1644 in the examined PCa cells is 4.1 μM, ranging between 2.1 μM and 6.3 μM (Figure 1C). Annexin V assay confirmed that BKM1644 inhibited PCa cell viability via the induction of apoptosis in a dose-dependent manner (Figure 1D). Taken together, these data indicate that BKM1644 exhibited potent *in vitro* cytotoxicity in PCa and other cancer cells.

### BKM1644 inhibits survivin transcription through a signal transducer and activator of transcription 3 (Stat3)-dependent mechanism

**Figure 2:**
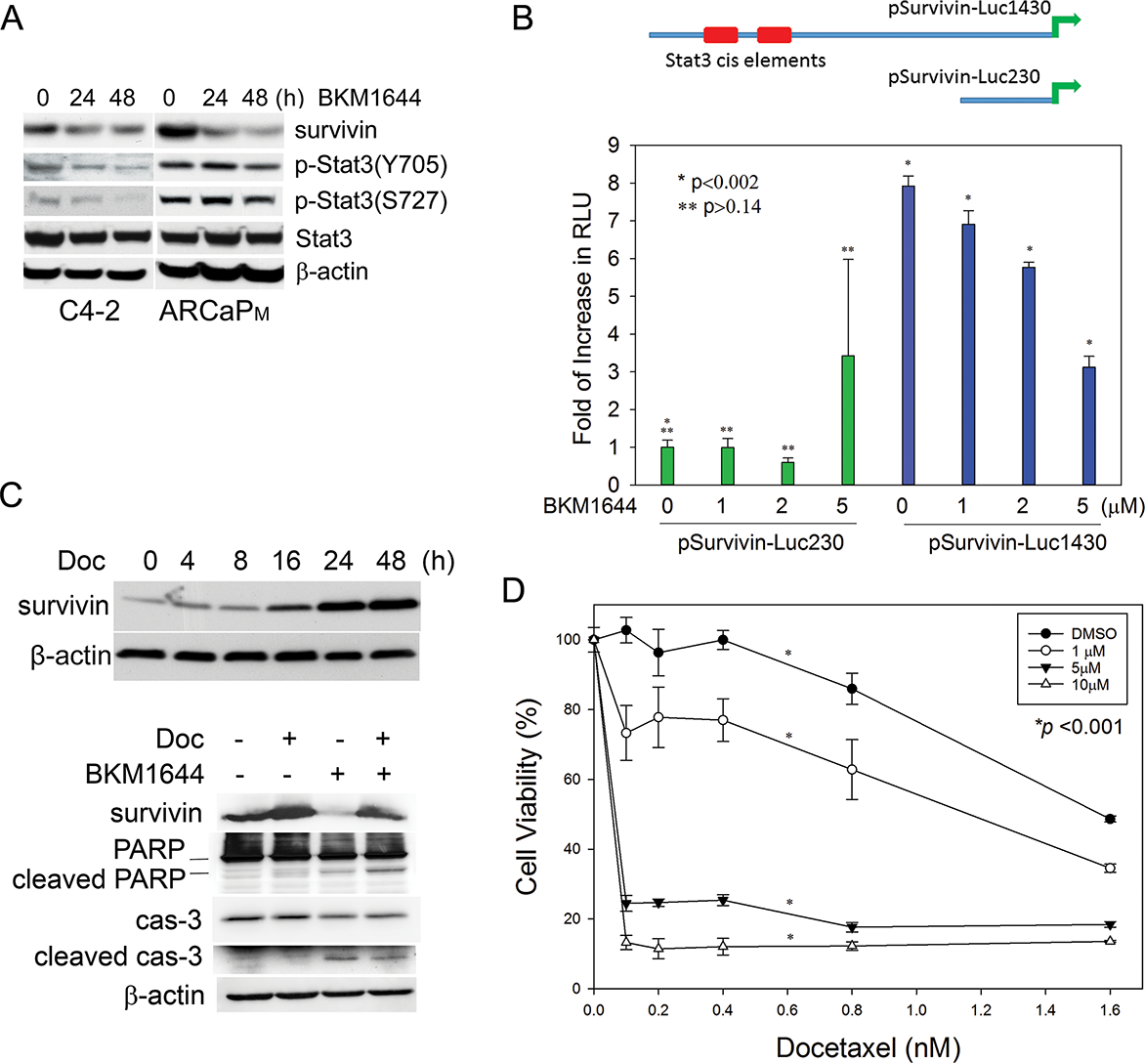
BKM1644 inhibits survivin expression through a Stat3-dependent mechanism (**A**) BKM1644 (5 μM) inhibits survivin proteins and the phosphorylation of Stat3 at Ser727 and Tyr705 residues in Western blot analysis. (**B**) Top panel: human survivin promoter contains two putative Stat3 binding elements; Bottom panel: BKM1644 selectively inhibits the luciferase activity of pSurvivin-Luc1430 in a dose-dependent manner. The reporters were transfected into C4-2 cells for 24 h prior to further treatment with BKM1644 for 48 h. (**C**) Top panel: Docetaxel treatment (2.5 nM) increases survivin protein expression in a time-dependent manner in C4-2 cells; Bottom panel: BKM1644 (5 μM) antagonizes survivin induction by docetaxel (2 nM) and activates apoptosis in C4-2 cells during a 24-h culture. (**D**) BKM1644 sensitizes C4-2 cells to docetaxel treatment in a dose-dependent manner (72 h).

Our previous studies identified BKM1740 as an inhibitor of survivin in PCa cells [13]. Given the structural similarity between BKM1740 and BKM1644, we examined whether BKM1644 also exerts an inhibitory effect on survivin expression. Indeed, BKM1644 effectively suppresses survivin proteins in a time-dependent manner in mCRPC cells (Figure 2A). To understand the mechanism of action of BKM1644 in PCa cells, we examined the effect of BKM1644 on several known upstream regulators of survivin [27–30] and found that Stat3 phosphorylation at both Tyr705 and Ser727 was significantly reduced upon BKM1644 treatment (Figure 2A). In fact, the human survivin promoter contains at least two Stat3 cis-elements, located between −1174 to −1166nt and between −1095 to −1087nt [31], that can be activated by the binding of phosphorylated Stat3 (Figure 2C, top panel). To determine whether Stat3 mediates the inhibitory effect of BKM1644 on survivin transcription, C4-2 cells were transfected with two luciferase reporters containing a 230-bp (pSurvivin-Luc230) or a 1430-bp fragment (pSurvivin-Luc1430) of the human survivin promoter, respectively [32]. Interestingly, the basal activity of pSurvivin-Luc1430, which contains the putative Stat3 cis-elements, was significantly higher (∼7.9 fold) than that of pSurvivin-Luc230, which does not contain the Stat3-binding sites. BKM1644 dose-dependently reduced the luciferase activity of pSurvivin-Luc1430 whereas it had a negligible effect on pSurvivin-Luc230 (Figure 2B). Taken together, these data indicated that BKM1644 inhibits survivin transcription through a Stat3-dependent mechanism.

### BKM1644 antagonizes docetaxel-induced survivin expression

Previously we observed that docetaxel treatment temporarily but significantly induces survivin expression in PCa cells, which may represent an underlying mechanism by which PCa cells acquire docetaxel resistance [33]. With the identification of BKM1644 as a survivin inhibitor, we further tested whether the presence of BKM1644 can antagonize the induction of survivin by docetaxel and sensitize PCa cells to docetaxel treatment. As shown in Figure 2C, top panel, docetaxel treatment resulted in a significant increase in survivin protein in C4-2 cells, starting at as early as 4 h and reaching a peak at 24 h. Simultaneous addition of BKM1644 to the cultures antagonized the induction of survivin and effectively promoted the cleavage of poly ADP ribose polymerase (PARP) and caspase 3, two indicators of apoptotic activation (Figure 2C, bottom panel). These data indicated that BKM1644 is capable of sensitizing PCa cells to docetaxel treatment via inhibition of survivin and induction of apoptosis. Supporting this notion, BKM1644 enhanced the *in vitro* cytotoxicity of docetaxel in a dose-dependent manner in mCRPC cells (Figure 2D).

### *In vivo* acute toxicity of BKM1644 in athymic nude mice

The acute toxicity of BKM1644 was evaluated by treating athymic nude mice with escalating doses of BKM1644 (10 mg/kg and 20 mg/kg, i.p, 3 times per week). Following a 4-week treatment, the body weights of both BKM1644 treatment groups continued to increase in a similar pattern to that in the vehicle control group (Figure S2). No obvious damage was observed in major organs. Blood analysis showed that only the dose of 20 mg/kg BKM1644 decreased the numbers of white blood cells (WBCs) and red blood cells (RBCs). The WBC and RBC counts in the 10 mg/kg group, and the kidney/liver function in both the 10 mg/kg and 20 mg/kg groups are within the normal range when compared to the control group (data not shown). Taken together, these results indicated that BKM1644 exhibits minimal acute toxicity and side effects when used at relatively high doses.

### BKM1644 inhibits the skeletal growth of C4-2 tumors in mice

**Figure 3:**
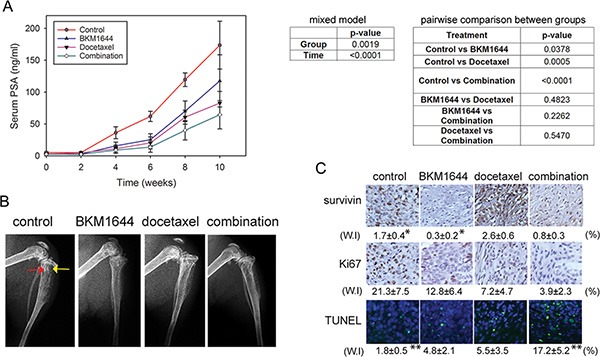
BKM1644 inhibits C4-2 tumor growth in mouse skeletons and enhances the *in vivo* efficacy of docetaxel (**A**) Left panel: BKM1644 alone or combined with docetaxel significantly reduces serum PSA levels in athymic nude mice bearing C4-2 tumors; Right panel: statistical analysis on the *in vivo* effect of BKM1644. (**B**) BKM1644 treatment improves the bone architecture in C4-2 tumor-bearing mice. Red arrow: osteoblastic lesion; yellow arrow: osteolytic lesion. (**C**) BKM1644 inhibits the *in vivo* expression of survivin and Ki67, and induces apoptosis in C4-2 skeletal tumor, as demonstrated by IHC and TUNEL assays. Two-tailed *t*-test was used to calculate *p* values on the weighted index (W.I). *, ** indicate *p*-values < 0.05.

We examined the *in vivo* efficacy of BKM1644 against the skeletal growth of mCRPC in animal models. C4-2 cells, which express high levels of androgen receptor and prostate-specific antigen (PSA), were inoculated into both tibias of male athymic nude mice. Four weeks after tumor inoculation, tumor-bearing mice were treated with vehicle control, docetaxel, BKM1644 or the combination of BKM1644 and docetaxel, respectively. Based on the acute toxicity results, a relatively low dose of 5 mg/kg BKM1644 was used in the efficacy experiment. Following a 10-week treatment, the average PSA level in each group at the endpoint was 173.72 ± 37.52 ng/ml (control), 82.77 ± 18.33 ng/ml (docetaxel), 117.67 ± 40.95 ng/ml (BKM1644), and 64.45 ± 22.19 ng/ml (BKM1644 and docetaxel combination), respectively (Figure 3A). Statistical analysis using a mixed model showed that there was a significant difference in the longitudinal PSA values across the 4 different treatment groups (*p* < 0.0019). The PSA value changes over time were also significant (*p* < 0.0001). The pairwise comparisons in PSA values over time between the two groups are summarized in Figure 3A, right panel. The BKM1644, docetaxel and the combination groups show a significant difference with the control group (all *p* < 0.05); however, they do not show significant differences among each other. Consistently, X-ray radiography showed that, compared with the control group, C4-2 tumor-bearing bone treated with either BKM1644, docetaxel or the combination regimen displayed improved architecture with reduced osteolytic destruction and osteoblastic lesions (Figure 3B), indicating an inhibitory effect of BKM1644 or the combination treatment on PCa growth in mouse bones.

### BKM1644 inhibits tissue expression of survivin and induces apoptosis in C4-2 skeletal tumor

The *in vivo* effect of the treatments on the expression of survivin and Ki67, a proliferation marker, was analyzed in C4-2 tumor specimens harvested at the endpoint (Figure 3C). Compared with the control group, docetaxel treatment resulted in an increase in survivin expression but reduced Ki67 levels. BKM1644 markedly reduced survivin (*p* = 0.035) and Ki67. The combination of BKM1644 and docetaxel inhibited survivin and Ki67, compared with the docetaxel treatment. Consistently, Terminal Deoxynucleotidyl Transferase dUTP Nick End Labeling (TUNEL) staining was higher in tumor tissues treated with BKM1644 or docetaxel, and further significantly increased in those from the combination group (*p* = 0.042) (Figure 3C). These data indicated that BKM1644 effectively inhibited survivin and induced apoptosis at the tissue level, which may contribute to the suppression of tumor growth in mouse bone and the enhanced efficacy of docetaxel chemotherapy.

## DISCUSSION

As a key pro-survival factor, survivin is overexpressed in most solid tumors [7]; therefore it has been actively pursued as a promising target to develop effective cancer therapy [34–36]. Various strategies are being explored, including vaccination strategies to generate an antigen-specific immune response against survivin-bearing tumor cells; the development of antisense oligonucleotides, ribozymes, or siRNA molecules targeting survivin; and small-molecule inhibitors of survivin function [37]. Among them, antisense oligonucleotides (LY2181308) and small-molecule inhibitors (YM155 and EM-1421) have entered clinical development. Treatments with YM155 have been well tolerated in general; however, only low to modest anti-tumor efficacy were reported [16, 37–39]. It remains challenging to develop survivin inhibitors to effectively enhance standard therapy and treat mCRPC.

Our previous work has shown an association between survivin overexpression with PCa bone metastasis [13], supporting survivin as a rational target to treat this lethal disease [14, 15]. We further developed a novel small-molecule survivin inhibitor BKM1740 and demonstrated its efficacy against bone metastatic PCa in pre-clinical models [13, 17]. In the current study, we report that BKM1644, an analog of BKM1740, exhibits potent anti-cancer activity in a broad spectrum of human cancer cells. We also provide evidence showing BKM1644 effectively targets survivin expression through a Stat3-dependent manner, and the combination of docetaxel and BKM1644 significantly inhibits the skeletal growth of mCRPC in mouse models. These results indicate that BKM1644 could be further evaluated as a promising small-molecule survivin inhibitor in pre-clinical and clinical settings.

Survivin expression in cancer cells is regulated at various levels independent of the cell cycle, including transcription, differential splicing, protein degradation, and intracellular sequestration [40, 41]. Several mechanisms have been identified, including amplification of the survivin locus, demethylation of survivin exons, increased promoter activity, and loss of function of p53. Other factors include activation of upstream signaling in the phosphatidylinositol 3-kinase (PI-3K)/Akt, mitogen-activated protein kinase (MAPK), Stat3, and Wnt signaling pathways [28–30]. As an effort to understand the mechanism of action of BKM1644 in PCa cells, we specifically examined the effect of BKM1644 on Stat3 signaling and found that this compound selectively inhibits the luciferase activity of pSurvivin-Luc1430, which contains at least two Stat3 cis-elements, suggesting a possible role of Stat3 as a mediator for the inhibitory effect of BKM1644 on survivin transcription. Although we will not exclude other possibilities, for example, BKM1644 may also affect survivin transcription through a PI-3K/Akt pathway or promote survivin degradation at post-translational levels [42], the data presented here clearly indicated that Stat3-mediated inhibition of survivin transcription could be an important mechanism of action of BKM1644 in inducing apoptosis in mCRPC cells.

Docetaxel remains the first-line chemotherapy for mCRPC [2, 3]. However, most patients develop therapeutic resistance, and tumors usually relapse after a short period of response. Multiple mechanisms may contribute to the acquired resistance to docetaxel, and among them, overexpression of survivin has been demonstrated to provide survival advantages in chemoresistant cancer cells [43, 44]. Several survivin inhibitors are being tested in clinical trials for their combination effects on docetaxel responsiveness. However, none of these trials has shown survival benefits. For example, a recent randomized phase 2 study combining LY2181308 with first-line docetaxel/prednisone failed to show efficacy in patients with mCRPC [45]. Among several possible reasons, insufficient inhibition of survivin by LY2181308, an antisense oligonucleotide with limited *in vivo* bioavailability and uptake in tumors, may contribute to its disappointing clinical efficacy [46]. In fact, it is estimated that an inhibition efficiency greater than 30% on survivin expression from baseline should be achieved to demonstrate the expected antitumor activity of the combined therapy of docetaxel and LY2181308 [45]. These results highlight a need to develop new survivin inhibitors with high potency and improved bioavailability profiles.

BKM1644 may have several advantages over currently available survivin-targeting agents. The design concept of BKM1644 and its analogs, including BKM1740, is to combine 2∼3 pharmacophoric subunits into one small molecule, with the expectation of achieving high anti-cancer activities. Specifically, these compounds incorporate two critical chemical structures: 1) F5c-OC2Y, the key moiety of BKM570; and 2) a bisphosphonate amide derivative (Figure 1A). In previous studies, we have shown that BKM570, one of our first-generation bradykinin antagonists, exhibited superior potency over cisplatin in inhibiting the *in vivo* growth of small-cell lung cancer xenografts [18–23]. Our structure-activity relationship assay demonstrated that the introduction of sterically hindered hydrophobic amino acid residues, such as OC2Y in BKM1644, could confer high anti-cancer efficacy [23]. The resulting compounds, including BKM1740 and BKM1644, effectively inhibit survivin expression at both the mRNA and protein levels *in vitro*, and significantly reduce survivin in bone metastatic C4-2 tumors [13, 17]. Further, the inclusion of an aminobisphosphonate moiety is expected to increase selective uptake of BKM compounds in bone tissues, thereby increasing their *in vivo* bioavailability and reducing systemic toxicity [26, 47]. Although this hypothesis still needs to be tested in future pharmacological studies, BKM1644 did exhibit a preferable safety profile and effectively retarded the skeletal growth of mCRPC when administered alone or combined with docetaxel. These results provide a strong rationale to further develop BKM1644 as a potent inhibitor of survivin and a lead compound for the management of PCa bone metastasis.

Consistent with our previous findings [33], an intriguing observation is that docetaxel treatment appears to increase survivin expression rapidly and significantly. Although the exact mechanism by which docetaxel increases survivin proteins remains unknown, two possibilities have been proposed in previous studies using paclitaxel, another widely used taxane drug. First, since survivin expression is cell cycle-regulated with a significant increase in the G2/M phase of the cell cycle [48, 49], cells treated with paclitaxel can result in an accumulation of survivin because of G2/M arrest [50]. Second, Liang et al. showed that in MCF-7 cells, the induction of survivin by paclitaxel is an early event (within 4 h at low doses) and independent of paclitaxel-mediated G2/M arrest. Instead, paclitaxel rapidly activated the PI-3K/Akt and MAPK pathways, which may subsequently activate survivin expression at both the transcriptional and post-transcriptional levels [51]. It is plausible to hypothesize that docetaxel may also use a similar mechanism in mCRPC cells, since we have repeatedly observed an early and rapid up-regulation of survivin proteins upon docetaxel treatment [33]. An aberrant accumulation of survivin could provide cancer cells an “emergent” protective mechanism and counteract docetaxel-induced apoptosis. Conversely, an agent that effectively antagonizes docetaxel-induced survivin could sensitize cancer cells to chemotherapy. Indeed, the presence of BKM1644 in cell cultures, as well as in C4-2 xenografts, attenuated docetaxel-induced induction of survivin, which could contribute to the observed efficacy of BKM1644 in retarding tumor growth in mouse bone and activating apoptosis *in vivo*, as evidenced by increased TUNEL staining in the combination group. Interestingly, although BKM1644 alone could effectively inhibit survivin in C4-2 cells, it only moderately reduced survivin expression in the presence of docetaxel. A possible cause might be that BKM1644 can only partially interfere with those signaling pathways activated by docetaxel treatment and responsible for survivin up-regulation in mCRPC cells. For example, in MCF-7 cells, paclitaxel potently activates a survivin promoter-luciferase construct containing a 2,840-bp sequence, but had no effect on pSurvivin-Luc1430, which may contain a major BKM1644 target region on the survivin promoter [51]. A close investigation on the mechanism by which docetaxel increases survivin can reveal other important players involved in the acquired resistance to docetaxel, and provide a rationale for combining BKM1644 and other agents to more effectively antagonize docetaxel-induced survivin expression and improve docetaxel chemotherapy.

A potential drawback in the reported clinical trials that failed to demonstrate therapeutic benefits of LY2181308 and YM155 is the lack of pre-screening of patients on their basal expression of survivin in tumors [45, 52]. Therefore it is difficult to conclude that the disappointing outcome is a result of inefficiency of these survivin inhibitors, or simply because the target (survivin) is not there. Future trials should identify and focus on patient subpopulations with elevated survivin levels in tumors. Given the prevalent expression of survivin in PCa bone metastasis [13–15] and the promising pre-clinical results presented here, BKM1644 could be developed as a novel small-molecule therapy to enhance standard chemotherapy and improve clinical outcomes in mCRPC patients.

## MATERIALS AND METHODS

### Synthesis of BKM1644 compound

BKM1644 was synthesized according to the procedure described by Gera et al. [17]. BKM1644 was submitted to and evaluated at the National Cancer Institute Developmental Therapeutics Program.

### Cell culture and reagents

Human PCa cell lines LNCaP, C4-2, C4-2B, CWR22Rv1, PC3, ARCaP_E_ and ARCaP_M_ were obtained from ATCC, or kindly provided by Dr. Leland WK Chung (Cedars Sinai Medical Center, Los Angeles, CA), and were routinely maintained in T-medium (Invitrogen, Carlsbad, CA) with 5% fetal bovine serum (FBS) or RPMI1640 medium supplemented with 10% FBS. Docetaxel (Taxotere^®^) was obtained from LC Laboratories (Woburn, MA).

### Cell proliferation assay

Cell proliferation was measured using the CellTiter 96 AQueous Non-Radioactive Cell Proliferation (MTS) Assay kit (Promega, Madison, WI) or Cell Counting Kit-8 (CCK-8; Dojindo Laboratories), according to the manufacturer's instruction. For the cell viability assay, 4 × 10^3^ cells per well were seeded on to 96-well plates overnight and treated with BKM1644, docetaxel or vehicle control at the indicated concentrations for 72 h. A microplate reader (Bio-Rad Laboratories, Hercules, CA) was used to determine cell viability, which was expressed as relative survival compared with controls recorded as 100%.

### Western blot analysis

Total cell lysates were prepared using radioimmunoprecipitation (RIPA) buffer (Santa Cruz Biotechnology). Immunoblotting analysis caspase-3, cleaved caspase-3 followed a standard procedure. Antibodies against PARP, phosphorylated Stat3 (p-Stat3, Ser727), and p-Stat3 (Tyr705) were purchased from Cell Signaling (Danvers, MA); survivin antibody was purchased from Novus Biologicals (Littleton, CO); β-actin antibody was purchased from Sigma-Aldrich (St. Louis, MO).

### Reporter assay

PCa cells were seeded at a density of 1 × 10^5^ cells per well in 24-well plates 24 h before transfection. Human survivin reporters (pSurvivin-Luc1430 and pSurvivin-Luc230; kindly provided by Dr. Allen Gao at University of California at Davis) were transfected with pRL-TK (internal control; Promega) using Lipofectamine 2000 (Invitrogen) following the manufacturer's instructions. Luciferase activities were measured at the indicated times using a Dual-Luciferase reporter assay system (Promega). Relative luciferase units were expressed as firefly luciferase intensity normalized to Renilla luciferase activity.

### Apoptosis analysis

Cells treated with DMSO or BKM1644 were trypsinized and washed with PBS and resuspended in Annexin-binding buffer (BD Pharmingen, San Diego, CA). Cells were then stained with both Annexin V-phycoerythrin and 7-amino-actinomycin for 15 min at room temperature. The stained samples for apoptosis assay were measured using a fluorescence-activated cell sorting (FACS) caliber bench-top flow cytometer (Becton Dickinson, Franklin Lakes, NJ). The data were analyzed using FlowJo software (Tree Star, Inc., Ashland, OR).

### *In vivo* acute toxicity

All animal procedures were performed in compliance with Emory University Institutional Animal Care and Use Committee (IACUC) and National Institutes of Health guidelines. A total of 12 athymic male nude mice (Hsd: athymic nude-nu; five-week-old; Harlan Laboratories, Indianapolis, IN) were randomized and evenly divided into 3 groups (4 mice per group) and treated for 4 weeks with vehicle control (100% DMSO) and BKM1644 at 10 mg/kg or 20 mg/kg of body weight via i.p route, 3 times per week. Mice were weighed every week and their behaviors were closely monitored. At endpoint, blood samples were collected through cardiac puncture, and subjected to complete blood count and blood chemistry for liver and kidney functions. Mice were sacrificed and major organs were closely examined for any abnormalities.

### Intratibial xenograft model

A total of 20 athymic male nude mice (Hsd: athymic nude-nu; five-week-old; Harlan Laboratories, Indianapolis, IN) were used. For each mouse, 1.0 × 10^6^ C4-2 cells were inoculated into the bilateral tibia using our established procedure [13]. Blood specimens were obtained from the facial veins every 2 weeks for serum PSA determination using an ELISA kit from United Biotech, Inc (Mountain View, CA). Four weeks later, tumor formation was confirmed by rising PSA levels. Tumor-bearing mice were randomly divided into four groups (5 mice per group), and mice received the following injection via the i.p. route for a 10-week period: vehicle control group: 100% DMSO, 3 times per week; docetaxel group: 5 mg/kg body weight, once per week; BKM1644: 5 mg/kg, 3 times per week; combination group: 5 mg/kg of docetaxel, once per week, and 5 mg/kg of BKM1644, 3 times per week. Mice were weighed every week, and tumor growth in bilateral tibia was followed by serum PSA and X-ray analyses with a Faxitron MX20 digital radiography system (Faxitron Bioptics, LLC; Tucson, AZ) every 2 weeks. At the end point, the bilateral tibia were removed, fixed in 10% neutralized formalin for 48 h, and decalcified in EDTA (pH 7.2) for 15 d. Tibia specimens were dehydrated and paraffin embedded at the Histomorphometry and Molecular Analysis Core, Department of Pathology, University of Alabama at Birmingham.

### Immunohistochemistry assay

Expression of survivin and Ki67 in C4-2 skeletal tumor tissues was analyzed by immunohistochemical staining using rabbit polyclonal antibodies against human survivin (Novus Biologicals; 1:300 dilution) and mouse monoclonal antibody against Ki67 (Dako; 1:100 dilution). Briefly, tissues were deparaffinized, rehydrated, and subjected to 5-min pressure-cooking antigen retrieval, 10-min double endogenous enzyme block, and overnight primary antibody incubation, and subjected to prediluted biotinylated pan-specific universal secondary antibody for 10 min. Signals were detected by adding 3, 3′-diaminobenzidine (DAB) substrate hydrogen peroxide and counterstained by hematoxylin QS. All reagents were obtained from Vector Laboratories (Burlingame, CA). Positive expression was defined as > 15% positive staining in the cell population. Weighted index was calculated (WI = % positive staining × intensity score [0, no expression; 2+, moderate expression; and 3+, strong expression]) to quantitate the relative intensity of IHC staining.

### Terminal deoxynucleotidyl transferase dUTP nick end labeling (TUNEL) assay

TUNEL assay was performed according to the manufacturer's instructions (TUNEL Apoptosis Detection Kit, GenScript, Piscataway, NJ). The slides were routinely dewaxed, hydrated, and then enzymatically digested with 20 μg/ml protease K for 30 min at room temperature. Slides then were washed in PBS and placed in 3% H_2_O_2_ in methanol for 10 min at room temperature. After washing in PBS, 50 μl TUNEL reaction mixture was added to the tissues and incubated for 60 min at 37°C. Slides were washed in PBS and 50 μl Streptavidin-HRP solution was added to the samples and incubated for 30 min at 37°C. After washing in PBS, DAB working solution was applied to the tissues for 3 min, then slides were routinely counterstained with hematoxylin and dehydrated for coverslipping with Permount.

### Statistical analysis

Treatment effects at specific time-points were evaluated using a two-sided Student's *t* test at each measurement time-point. To assess the longitudinal effect of treatment, a mixed model was employed to test the overall difference across all groups as well as between each pair of groups during the whole study period. The significance levels were set at 0.05 for all tests. The SAS statistical package V9.2 (SAS Institute, Inc., Cary, North Carolina) was used for data management and analysis.

## SUPPLEMENTARY MATERIALS FIGURES



## Footnotes

a BKM1740: CAS Registry Number 1070966-97-0 *P*,*P*′-[[[1-[(2*S*)-3-[4-[(2,6-Dichlorophenyl)methoxy]phenyl]-1-oxo-2-[[1-oxo-3-(2,3,4,5,6-pentafluorophenyl)-2-propen-1-yl]amino]propyl]-4-piperidinyl]amino]methylene] bis-phosphonic acid *P*,*P*,*P*′,*P*′-tetraethyl ester; C_39_H_46_ Cl_2_F_5_N_3_O_9_P_2_ = 928.64.

b BKM1644: CAS Registry Number 1070966-96-9. *P*,*P*′-[[[(2*S*)-3-[4-[(2,6-Dichlorophenyl)methoxy]phenyl]-1-oxo-2-[[1-oxo-3-(2,3,4,5,6-pentafluorophenyl)-2-propen-1-yl]amino]propyl]amino]methylene]bis-phosphonic acid *P*,*P*,*P*′,*P*′-tetraethyl ester; C_34_H_37_Cl_2_F_5_N_2_O_9_P_2_ = 845.51.

## Abbreviations

AMDP(OEt)_4_: tetraethyl aminomethylenediphosphonate
F5c: 2,3,4,5,6-pentafluorocinnamoyl
GI_50_: 50% growth inhibition
LC_50_: 50% lethal concentration
mCRPC: metastatic, castration-resistant prostate cancer
OC2Y: O-(2,6-dichlorobenzyl)tyrosyl
PARP: poly ADP ribose polymerase
PCa: prostate cancer
PSA: prostate-specific antigen
Stat3: signal transducers and activators of transcription 3
TUNEL: Terminal Deoxynucleotidyl Transferase dUTP Nick End Labeling

## References

[1] Siegel RL, Miller KD, Jemal A (2016). Cancer statistics, 2016. CA Cancer J Clin.

[2] Petrylak DP, Tangen CM, Hussain MH, Lara PN, Jones JA, Taplin ME, Burch PA, Berry D, Moinpour C, Kohli M, Benson MC, Small EJ, Raghavan D (2004). Docetaxel and estramustine compared with mitoxantrone and prednisone for advanced refractory prostate cancer. N Engl J Med.

[3] Tannock IF, de Wit R, Berry WR, Horti J, Pluzanska A, Chi KN, Oudard S, Theodore C, James ND, Turesson I, Rosenthal MA, Eisenberger MA (2004). Docetaxel plus prednisone or mitoxantrone plus prednisone for advanced prostate cancer. N Engl J Med.

[4] van Dodewaard-de Jong JM, Verheul HM, Bloemendal HJ, de Klerk JM, Carducci MA, van den Eertwegh AJ (2015). New Treatment Options for Patients With Metastatic Prostate Cancer: What Is The Optimal Sequence?. Clin Genitourin Cancer.

[5] Agarwal N, Di Lorenzo G, Sonpavde G, Bellmunt J (2014). New agents for prostate cancer. Ann Oncol.

[6] Omlin A, Pezaro C, Gillessen Sommer S (2014). Sequential use of novel therapeutics in advanced prostate cancer following docetaxel chemotherapy. Ther Adv Urol.

[7] Altieri DC (2013). Targeting survivin in cancer. Cancer Lett.

[8] Rivadeneira DB, Caino MC, Seo JH, Angelin A, Wallace DC, Languino LR, Altieri DC (2015). Survivin promotes oxidative phosphorylation, subcellular mitochondrial repositioning, and tumor cell invasion. Sci Signal.

[9] Kishi H, Igawa M, Kikuno N, Yoshino T, Urakami S, Shiina H (2004). Expression of the survivin gene in prostate cancer: correlation with clinicopathological characteristics, proliferative activity and apoptosis. J Urol.

[10] Shariat SF, Lotan Y, Saboorian H, Khoddami SM, Roehrborn CG, Slawin KM, Ashfaq R (2004). Survivin expression is associated with features of biologically aggressive prostate carcinoma. Cancer.

[11] Nomura T, Yamasaki M, Nomura Y, Mimata H (2005). Expression of the inhibitors of apoptosis proteins in cisplatin-resistant prostate cancer cells. Oncol Rep.

[12] Zhang M, Latham DE, Delaney MA, Chakravarti A (2005). Survivin mediates resistance to antiandrogen therapy in prostate cancer. Oncogene.

[13] Seo SI, Gera L, Zhau HE, Qian WP, Iqbal S, Johnson NA, Zhang S, Zayzafoon M, Stewart J, Wang R, Chung LW, Wu D (2008). BKM1740, an acyl-tyrosine bisphosphonate amide derivative, inhibits the bone metastatic growth of human prostate cancer cells by inducing apoptosis. Clin Cancer Res.

[14] Zhang M, Coen JJ, Suzuki Y, Siedow MR, Niemierko A, Khor LY, Pollack A, Zhang Y, Zietman AL, Shipley WU, Chakravarti A (2010). Survivin is a potential mediator of prostate cancer metastasis. Int J Radiat Oncol Biol Phys.

[15] Akfirat C, Zhang X, Ventura A, Berel D, Colangelo ME, Miranti CK, Krajewska M, Reed JC, Higano CS, True LD, Vessella RL, Morrissey C, Knudsen BS (2013). Tumour cell survival mechanisms in lethal metastatic prostate cancer differ between bone and soft tissue metastases. J Pathol.

[16] Tolcher AW, Quinn DI, Ferrari A, Ahmann F, Giaccone G, Drake T, Keating A, de Bono JS (2012). A phase II study of YM155, a novel small-molecule suppressor of survivin, in castration-resistant taxane-pretreated prostate cancer. Ann Oncol.

[17] Gera L, Stewart J, Chung LW, Wu D (2008). Compositions and methods for treating bone cancer. PCT Int Appl WO.

[18] Gera L, Chan DC, Helfrich B, Bunn J, P. A., York EJ, Stewart JM, Martinez J, Fehrentz JA (2001). Bradykinin-related compounds having anti-cancer activity *in vivo* superior to Cisplatin and SU5416. Peptides 2000.

[19] Stewart JM (2003). Bradykinin antagonists as anti-cancer agents. Current pharmaceutical design.

[20] Stewart JM (2004). Bradykinin antagonists: discovery and development. Peptides.

[21] Stewart JM, Chan DC, Simkeviciene V, Bunn PA, Helfrich B, York EJ, Taraseviciene-Stewart L, Bironaite D, Gera L (2002). Bradykinin antagonists as new drugs for prostate cancer. Int Immunopharmacol.

[22] Stewart JM, Gera L, Chan DC, York EJ, Simkeviciene V, Bunn PA, Taraseviciene-Stewart L (2005). Combination cancer chemotherapy with one compound: pluripotent bradykinin antagonists. Peptides.

[23] Stewart JM, Gera L, Chan DC, Bunn PA, York EJ, Simkeviciene V, Helfrich B (2002). Bradykinin-related compounds as new drugs for cancer and inflammation. Can J Physiol Pharmacol.

[24] Dunford JE, Thompson K, Coxon FP, Luckman SP, Hahn FM, Poulter CD, Ebetino FH, Rogers MJ (2001). Structure-activity relationships for inhibition of farnesyl diphosphate synthase *in vitro* and inhibition of bone resorption *in vivo* by nitrogen-containing bisphosphonates. J Pharmacol Exp Ther.

[25] Saad F, Gleason DM, Murray R, Tchekmedyian S, Venner P, Lacombe L, Chin JL, Vinholes JJ, Goas JA, Chen B, Zoledronic Acid Prostate Cancer Study G (2002). A randomized, placebo-controlled trial of zoledronic acid in patients with hormone-refractory metastatic prostate carcinoma. J Natl Cancer Inst.

[26] Lin JH (1996). Bisphosphonates: a review of their pharmacokinetic properties. Bone.

[27] Kanda N, Seno H, Konda Y, Marusawa H, Kanai M, Nakajima T, Kawashima T, Nanakin A, Sawabu T, Uenoyama Y, Sekikawa A, Kawada M, Suzuki K (2004). STAT3 is constitutively activated and supports cell survival in association with survivin expression in gastric cancer cells. Oncogene.

[28] Mahboubi K, Li F, Plescia J, Kirkiles-Smith NC, Mesri M, Du Y, Carroll JM, Elias JA, Altieri DC, Pober JS (2001). Interleukin-11 up-regulates survivin expression in endothelial cells through a signal transducer and activator of transcription-3 pathway. Lab Invest.

[29] Vaira V, Lee CW, Goel HL, Bosari S, Languino LR, Altieri DC (2007). Regulation of survivin expression by IGF-1/mTOR signaling. Oncogene.

[30] You L, He B, Xu Z, Uematsu K, Mazieres J, Mikami I, Reguart N, Moody TW, Kitajewski J, McCormick F, Jablons DM (2004). Inhibition of Wnt-2-mediated signaling induces programmed cell death in non-small-cell lung cancer cells. Oncogene.

[31] Gritsko T, Williams A, Turkson J, Kaneko S, Bowman T, Huang M, Nam S, Eweis I, Diaz N, Sullivan D, Yoder S, Enkemann S, Eschrich S (2006). Persistent activation of stat3 signaling induces survivin gene expression and confers resistance to apoptosis in human breast cancer cells. Clin Cancer Res.

[32] Li F, Altieri DC (1999). Transcriptional analysis of human survivin gene expression. Biochem J.

[33] Wang Y, Zhang S, Iqbal S, Chen Z, Wang X, Wang YA, Liu D, Bai K, Ritenour C, Kucuk O, Wu D (2013). Pomegranate extract inhibits the bone metastatic growth of human prostate cancer cells and enhances the *in vivo* efficacy of docetaxel chemotherapy. Prostate.

[34] Xiao M, Li W (2015). Recent Advances on Small-Molecule Survivin Inhibitors. Curr Med Chem.

[35] Coumar MS, Tsai FY, Kanwar JR, Sarvagalla S, Cheung CH (2013). Treat cancers by targeting survivin: just a dream or future reality?. Cancer Treat Rev.

[36] Church DN, Talbot DC (2012). Survivin in solid tumors: rationale for development of inhibitors. Curr Oncol Rep.

[37] Altieri DC (2008). Survivin, cancer networks and pathway-directed drug discovery. Nat Rev Cancer.

[38] Tolcher AW, Hao D, de Bono J, Miller A, Patnaik A, Hammond LA, Smetzer L, Van Wart Hood J, Merritt J, Rowinsky EK, Takimoto C, Von Hoff D, Eckhardt SG (2006). Phase I, pharmacokinetic, and pharmacodynamic study of intravenously administered Ad5CMV-p53, an adenoviral vector containing the wild-type p53 gene, in patients with advanced cancer. J Clin Oncol.

[39] Tuma RS (2009). Agents targeting apoptosis show promise in early trials. J Natl Cancer Inst.

[40] Altieri DC (2004). Molecular circuits of apoptosis regulation and cell division control: the survivin paradigm. J Cell Biochem.

[41] Boidot R, Vegran F, Lizard-Nacol S (2014). Transcriptional regulation of the survivin gene. Mol Biol Rep.

[42] Liu YB, Gao X, Deeb D, Brigolin C, Zhang Y, Shaw J, Pindolia K, Gautam SC (2014). Ubiquitin-proteasomal degradation of antiapoptotic survivin facilitates induction of apoptosis in prostate cancer cells by pristimerin. Int J Oncol.

[43] Singh N, Krishnakumar S, Kanwar RK, Cheung CH, Kanwar JR (2015). Clinical aspects for survivin: a crucial molecule for targeting drug-resistant cancers. Drug Discov Today.

[44] Al-Dimassi S, Abou-Antoun T, El-Sibai M (2014). Cancer cell resistance mechanisms: a mini review. Clin Transl Oncol.

[45] Wiechno P, Somer BG, Mellado B, Chlosta PL, Cervera Grau JM, Castellano D, Reuter C, Stockle M, Kamradt J, Pikiel J, Duran I, Wedel S, Callies S (2014). A randomised phase 2 study combining LY2181308 sodium (survivin antisense oligonucleotide) with first-line docetaxel/prednisone in patients with castration-resistant prostate cancer. Eur Urol.

[46] Talbot DC, Ranson M, Davies J, Lahn M, Callies S, Andre V, Kadam S, Burgess M, Slapak C, Olsen AL, McHugh PJ, de Bono JS, Matthews J (2010). Tumor survivin is downregulated by the antisense oligonucleotide LY2181308: a proof-of-concept, first-in-human dose study. Clin Cancer Res.

[47] Saad F, Gleason DM, Murray R, Tchekmedyian S, Venner P, Lacombe L, Chin JL, Vinholes JJ, Goas JA, Chen B (2002). A randomized, placebo-controlled trial of zoledronic acid in patients with hormone-refractory metastatic prostate carcinoma. J Natl Cancer Inst.

[48] Li F, Ambrosini G, Chu EY, Plescia J, Tognin S, Marchisio PC, Altieri DC (1998). Control of apoptosis and mitotic spindle checkpoint by survivin. Nature.

[49] Li F, Altieri DC (1999). The cancer antiapoptosis mouse survivin gene: characterization of locus and transcriptional requirements of basal and cell cycle-dependent expression. Cancer Res.

[50] O'Connor DS, Wall NR, Porter AC, Altieri DC (2002). A p34(cdc2) survival checkpoint in cancer. Cancer Cell.

[51] Ling X, Bernacki RJ, Brattain MG, Li F (2004). Induction of survivin expression by taxol (paclitaxel) is an early event, which is independent of taxol-mediated G2/M arrest. J Biol Chem.

[52] Kelly RJ, Thomas A, Rajan A, Chun G, Lopez-Chavez A, Szabo E, Spencer S, Carter CA, Guha U, Khozin S, Poondru S, Van Sant C, Keating A (2013). A phase I/II study of sepantronium bromide (YM155, survivin suppressor) with paclitaxel and carboplatin in patients with advanced non-small-cell lung cancer. Ann Oncol.

