# Supplementary material for: Inhibition of skeletal growth of human prostate cancer by the combination of docetaxel and BKM1644: an aminobisphosphonate derivative
Source: Oncotarget. 2016 Mar 30;7(19):27489–98. doi: 10.18632/oncotarget.8481 (PMC5053666; doi:10.18632/oncotarget.8481)
Supplement: Supplementary file 1 [file oncotarget-07-27489-s001.pdf]

Inhibition of skeletal growth of human prostate cancer by the combination of docetaxel and BKM1644, an aminobisphosphonate derivative

Supplementary Materials

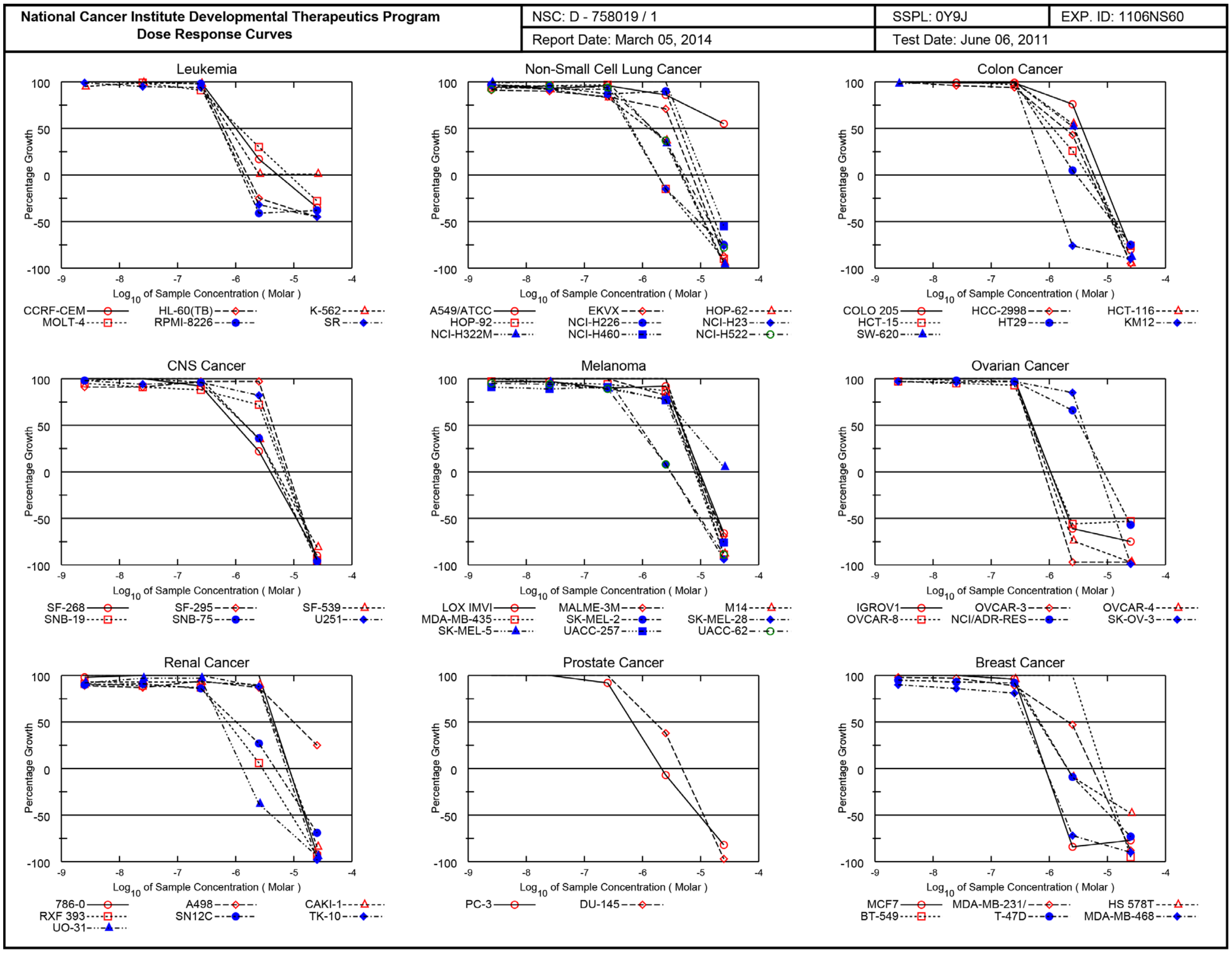

National Cancer Institute Developmental Therapeutics Program
In-Vitro Testing Results

NSC : D - 758019 / 1	Experiment ID : 1106NS60	Test Type : 08	Units : Molar
Report Date : March 05, 2014	Test Date : June 06, 2011	QNS :	MC :
COMI : GH1002 (103735)	Stain Reagent : SRB Dual-Pass Related	SSPL : 0Y9J	

	Time		Log10 Concentration													
			Mean Optical Densities					Percent Growth								
Panel/Cell Line	Zero	Ctrl	-8.6	-7.6	-6.6	-5.6	-4.6	-8.6	-7.6	-6.6	-5.6	-4.6	GI50	TGI	LC50
**Leukemia**															
CCRF-CEM	0.506	1.646	1.658	1.715	1.677	0.705	0.329	101	106	103	17	-35	1.04E-6	5.37E-6	> 2.50E-5
HL-60(TB)	1.198	3.257	3.269	3.287	3.307	0.897	0.660	101	101	102	-25	-45	6.44E-7	1.59E-6	> 2.50E-5
K-562	0.284	1.823	1.752	1.812	1.798	0.296	0.296	95	99	98	1	1	7.83E-7	> 2.50E-5	> 2.50E-5
MOLT-4	0.632	2.447	2.457	2.432	2.277	1.181	0.458	101	99	91	30	-28	1.18E-6	8.35E-6	> 2.50E-5
RPMI-8226	0.798	1.803	1.891	1.874	1.779	0.469	0.491	109	107	98	-41	-38	5.50E-7	1.26E-6	> 2.50E-5
SR	0.695	2.674	2.651	2.572	2.555	0.471	0.381	99	95	94	-32	-45	5.57E-7	1.39E-6	> 2.50E-5
**Non-Small Cell Lung Cancer**															
A549/ATCC	0.407	1.863	1.826	1.764	1.802	1.657	1.207	97	93	96	86	55	> 2.50E-5	> 2.50E-5	> 2.50E-5
EKVX	0.641	1.497	1.422	1.408	1.359	1.249	0.081	91	90	84	71	-87	3.39E-6	7.02E-6	1.45E-5
HOP-62	0.462	1.228	1.190	1.168	1.098	0.744	0.030	95	92	83	37	-94	1.30E-6	4.79E-6	1.16E-5
HOP-92	1.092	1.311	1.299	1.303	1.305	0.934	0.113	94	96	97	-15	-90	6.62E-7	1.85E-6	7.41E-6
NCI-H226	0.680	1.411	1.387	1.373	1.319	1.337	0.173	97	95	87	90	-75	4.37E-6	8.80E-6	1.77E-5
NCI-H23	0.598	1.779	1.734	1.686	1.686	0.506	0.145	96	92	92	-15	-76	6.16E-7	1.80E-6	9.36E-6
NCI-H322M	0.709	1.464	1.456	1.476	1.487	0.962	0.026	99	102	103	34	-96	1.45E-6	4.53E-6	1.10E-5
NCI-H460	0.249	1.776	1.954	1.985	1.887	1.779	0.112	112	114	107	100	-55	5.26E-6	1.10E-5	2.32E-5
NCI-H522	0.809	1.792	1.714	1.748	1.733	1.171	0.179	92	96	94	37	-78	1.47E-6	5.23E-6	1.43E-5
**Colon Cancer**															
COLO 205	0.222	0.983	1.005	0.974	0.975	0.800	0.044	103	99	99	76	-80	3.66E-6	7.66E-6	1.60E-5
HCC-2998	0.823	2.606	2.606	2.528	2.505	1.586	0.045	100	96	94	43	-95	1.81E-6	5.12E-6	1.18E-5
HCT-116	0.183	1.207	1.249	1.205	1.257	0.750	0.010	104	100	105	55	-95	2.71E-6	5.84E-6	1.26E-5
HCT-15	0.445	2.272	2.266	2.310	2.225	0.926	0.105	100	102	97	26	-76	1.16E-6	4.51E-6	1.38E-5
HT29	0.230	1.079	1.163	1.186	1.210	0.273	0.057	110	113	115	5	-75	9.79E-7	2.89E-6	1.21E-5
KM12	0.339	1.398	1.441	1.457	1.439	0.080	0.034	104	106	104	-76	-90	4.97E-7	9.42E-7	1.78E-6
SW-620	0.238	1.462	1.443	1.496	1.465	0.872	0.029	98	103	100	52	-88	2.57E-6	5.87E-6	1.34E-5
**CNS Cancer**															
SF-268	0.506	1.559	1.570	1.571	1.477	0.735	0.053	101	101	92	22	-90	9.93E-7	3.92E-6	1.10E-5
SF-295	0.745	2.138	2.020	2.019	2.095	2.094	0.040	91	91	97	97	-95	4.39E-6	8.01E-6	1.46E-5
SF-539	0.765	1.854	1.939	1.849	1.964	1.149	0.145	108	100	110	35	-81	1.59E-6	5.02E-6	1.35E-5
SNB-19	0.463	1.436	1.389	1.348	1.319	1.162	0.022	95	91	88	72	-95	3.38E-6	6.73E-6	1.34E-5
SNB-75	0.564	1.100	1.089	1.108	1.081	0.755	0.025	98	102	96	36	-96	1.45E-6	4.67E-6	1.12E-5
U251	0.331	1.461	1.439	1.392	1.421	1.260	0.016	98	94	96	82	-95	3.80E-6	7.26E-6	1.39E-5
**Melanoma**															
LOX IMVI	0.305	1.775	1.725	1.725	1.621	1.658	0.104	97	97	90	92	-66	4.61E-6	9.55E-6	1.98E-5
MALME-3M	0.719	1.491	1.506	1.506	1.515	1.350	0.222	102	102	103	82	-69	4.05E-6	8.69E-6	1.87E-5
M14	0.388	1.267	1.290	1.275	1.288	1.273	0.048	103	101	102	101	-88	4.64E-6	8.55E-6	1.58E-5
MDA-MB-435	0.424	1.836	1.787	1.775	1.753	1.656	0.046	97	96	94	87	-89	4.06E-6	7.80E-6	1.50E-5
SK-MEL-2	1.029	1.798	1.884	1.928	1.912	1.855	0.251	111	117	115	107	-76	5.14E-6	9.65E-6	1.81E-5
SK-MEL-28	0.486	1.148	1.157	1.183	1.157	0.536	0.031	101	105	101	8	-94	8.82E-7	2.97E-6	9.25E-6
SK-MEL-5	0.445	2.557	2.565	2.473	2.332	2.100	0.555	100	96	89	78	5	6.10E-6	> 2.50E-5	> 2.50E-5
UACC-257	0.690	1.427	1.358	1.343	1.359	1.254	0.168	91	89	91	77	-76	3.73E-6	7.96E-6	1.70E-5
UACC-62	0.749	2.228	2.153	2.134	2.065	0.861	0.086	95	94	89	8	-89	7.53E-7	3.00E-6	9.92E-6
**Ovarian Cancer**															
IGROV1	0.428	1.386	1.387	1.479	1.444	0.169	0.107	100	110	106	-61	-75	5.42E-7	1.08E-6	2.16E-6
OVCAR-3	0.403	1.115	1.143	1.147	1.194	0.014	0.013	104	104	111	-97	-97	4.92E-7	8.57E-7	1.49E-6
OVCAR-4	0.609	1.502	1.584	1.528	1.553	0.160	0.019	109	103	106	-74	-97	5.11E-7	9.70E-7	1.84E-6
OVCAR-8	0.365	1.385	1.354	1.336	1.317	0.161	0.172	97	95	93	-56	-53	4.88E-7	1.05E-6	2.28E-6
NCI/ADR-RES	0.487	1.465	1.525	1.447	1.437	1.136	0.209	106	98	97	66	-57	3.39E-6	8.61E-6	2.19E-5
SK-OV-3	0.533	1.259	1.236	1.228	1.237	1.147	0.008	97	96	97	85	-99	3.86E-6	7.24E-6	1.36E-5
**Renal Cancer**															
786-0	0.572	2.014	1.983	2.016	2.085	2.172	0.044	98	100	105	111	-92	4.99E-6	8.78E-6	1.55E-5
A498	1.260	2.004	1.921	1.909	1.963	1.907	1.443	89	87	94	87	25	9.79E-6	> 2.50E-5	> 2.50E-5
CAKI-1	0.687	2.159	2.052	2.056	2.060	2.008	0.113	93	93	93	90	-84	4.24E-6	8.23E-6	1.60E-5
RXF 393	0.635	1.197	1.144	1.133	1.121	0.671	0.038	91	89	87	6	-94	7.14E-7	2.89E-6	9.10E-6
SN12C	0.590	1.903	1.773	1.782	1.717	0.945	0.183	90	91	86	27	-69	1.02E-6	4.78E-6	1.58E-5
TK-10	0.702	1.203	1.223	1.211	1.217	1.144	0.013	104	101	103	88	-98	4.01E-6	7.43E-6	1.38E-5
UO-31	0.552	1.740	1.643	1.710	1.706	0.340	0.032	92	97	97	-38	-94	5.56E-7	1.30E-6	4.03E-6
**Prostate Cancer**															
PC-3	0.595	1.545	1.546	1.552	1.466	0.551	0.106	100	101	92	-7	-82	6.58E-7	2.10E-6	9.27E-6
DU-145	0.449	1.373	1.409	1.421	1.461	0.797	0.015	104	105	109	38	-97	1.68E-6	4.77E-6	1.12E-5
**Breast Cancer**															
MCF7	0.402	1.621	1.640	1.649	1.575	0.064	0.091	102	102	96	-84	-77	4.51E-7	8.54E-7	1.62E-6
MDA-MB-231/ATCC	0.581	1.421	1.401	1.399	1.332	0.979	0.071	98	97	89	47	-88	2.16E-6	5.60E-6	1.31E-5
HS 578T	0.841	1.491	1.514	1.554	1.467	0.767	0.436	104	110	96	-9	-48	6.90E-7	2.06E-6	> 2.50E-5
BT-549	0.821	1.525	1.613	1.561	1.674	1.633	0.039	112	105	121	115	-95	5.11E-6	8.82E-6	1.52E-5
T-47D	0.722	1.563	1.525	1.505	1.493	0.658	0.199	95	93	92	-9	-73	6.50E-7	2.04E-6	1.11E-5
MDA-MB-468	0.606	1.401	1.318	1.287	1.252	0.169	0.064	90	86	81	-72	-90	3.99E-7	8.46E-7	1.79E-6

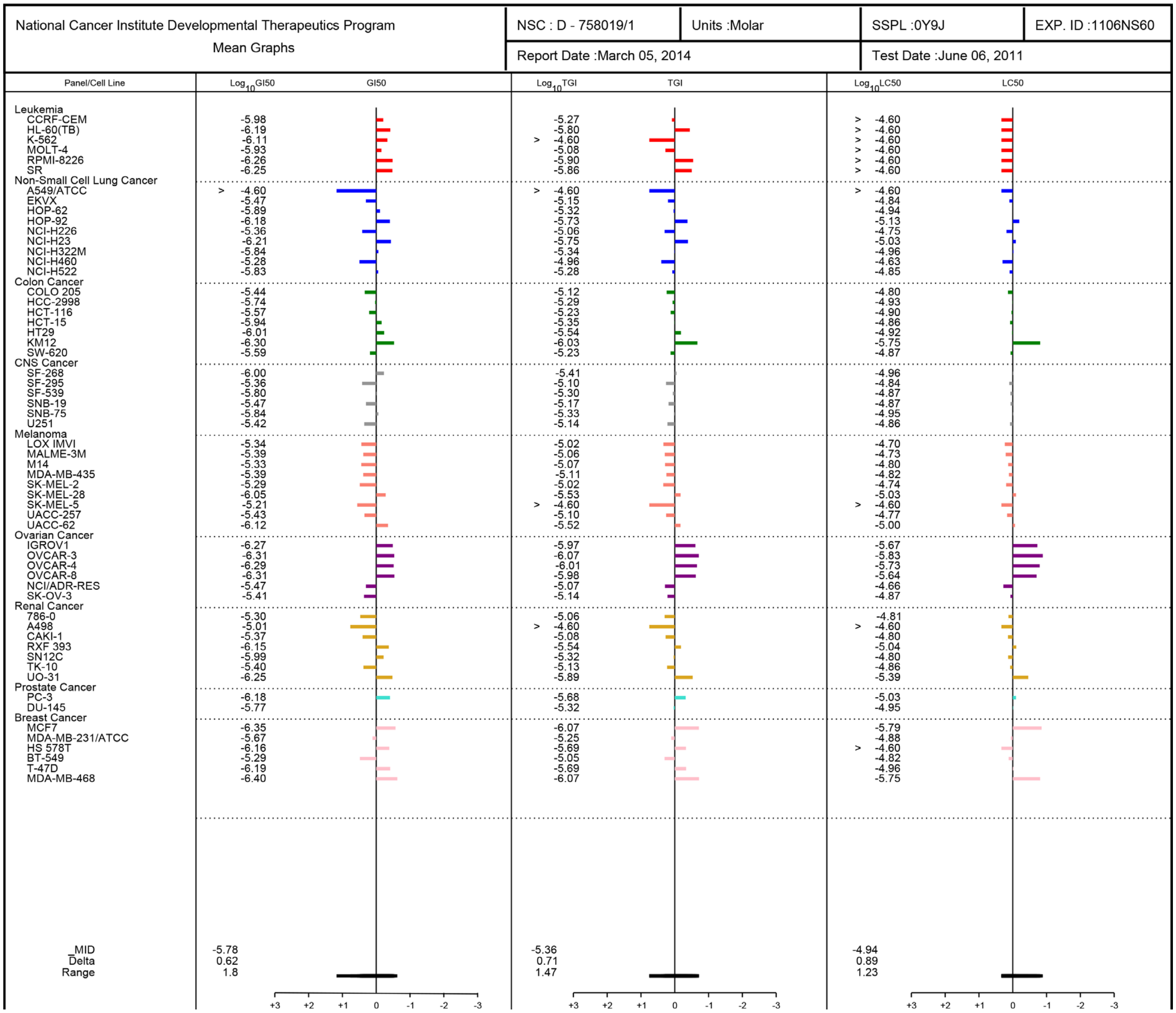

Supplementary Figure S1: *In vitro* activity of BKM1644 in NCI-60 panel. Compound activities presented as GI50, TGI (total growth inhibition) and LC50, using the median value as the y-axis, respectively.

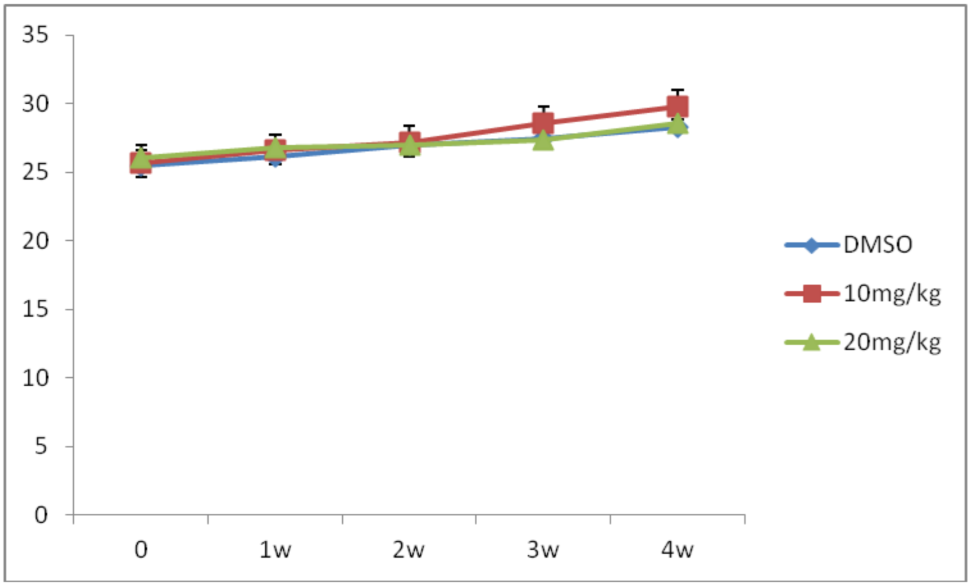

Supplementary Figure S2: Body weight of mice treated with BKM1644 at 10 mg/kg and 20 mg/kg, and the vehicle control (DMSO) via i.p. route, 3 times per week for 4 weeks. *n* = 4 per group.
